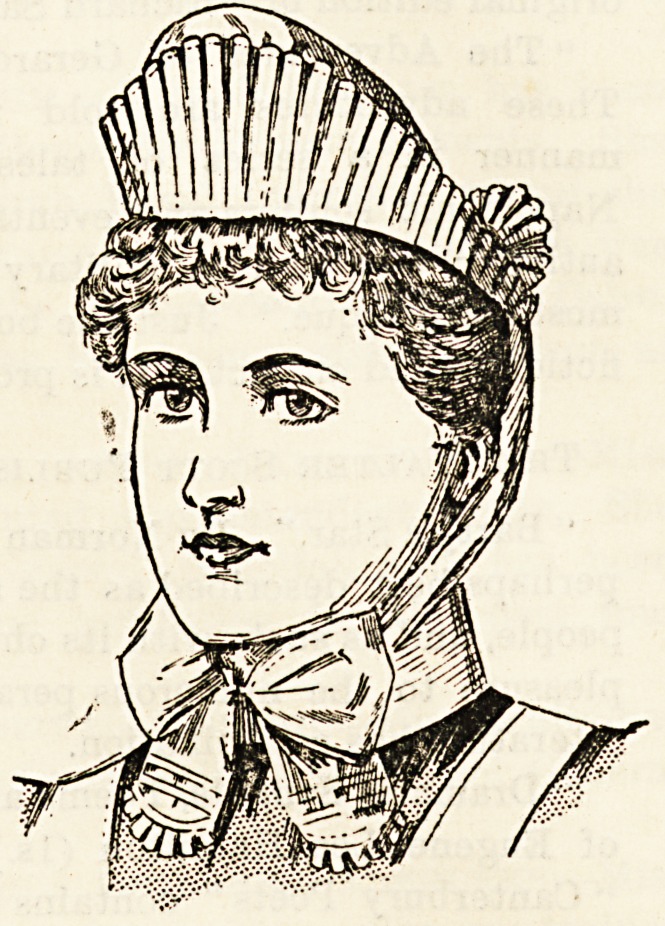# Nursing Section

**Published:** 1903-12-19

**Authors:** 


### The Hospital.

### Nursing Section.

Contributions for this Section of "The Hospital" should be addressed to the Editor, "The Hospital" Nursing Section, 28 & 29 Southampton Street, Strand, London, W.C

No. 899.—Vol. XXXV. *SATURDAY, DECEMBER* 19, 1903.

### Notes on News from the Nursing World.

### OUR CHRISTMAS DISTRIBUTION.

The articles so kindly sent by our readers for our Christmas distribution were on view at the offices of The Hospital on Tuesday afternoon. They made a goodly show when they were set out, and afforded the best possible evidence that their makers well understood the needs they so thoughtfully supplied. The majority of the articles were made of wool or flannel, and included two pretty little knitted baby hoods. There was an admirable variety of garments, but all were useful and many also ornamental, notably the dainty pinafores and little frocks. As might be expected, a large proportion of the work had been done on behalf of children, although the adults, both male and female, were by no means overlooked. When the exhibition closed the gifts were apportioned to various hospitals and infirmaries—of which a list will be published next week—and were duly dispatched in good time to their destinations.

### CHRISTMAS PREPARATIONS AT THE LONDON HOSPITALS.

Christmas will be observed in the good old-fashioned style at the London hospitals. At the London, the day will be ushered in by carol-singing by the sisters and nurses, who will visit each ward in turn; presents will be distributed by Father Christmas; turkeys, roast beef, and plum pudding will form the dinner for those patients who are well enough to be on full diet, and an elaborate system of entertainments is being organised by doctors and students. At Guy's, a leading feature will be the entertainment to out-patients, where hundreds of guests are expected; and ward concerts are also being arranged. The choir boys of St. Martin's will, according to their custom, sing carols in the wards of Charing Cross Hospital, which will be prettily decorated with fairy lamps, paper chains, ivy, etc., the men will smoke, and there will be ward concerts in the evening, presents having been distributed by the night nurses early in the day. Each ward sister has received a guinea from a member of the committee towards the expenses of the Christmas tea, and a collection for the same object has been made in the board-room. The Royal Free Hospital will be honoured on January 13th by a visit from the President, Princess Christian, who will give a tree for nurses and patients in Calthorpe ward, and who will afterwards go through the hospital to distribute her gifts to those patients who are confined to bed. The carol-singing procession by students with coloured lamps will take place as usual on Christmas Eve, and the patients will have their friends to tea on the afternoon of Christmas Day. At King's College Hospital, the 28th is the date selected for the "Boxing Day Entertainment," and on Christmas Day itself there will be good old English fare and festive teas. All the entertainments at Westminster Hospital will be on Christmas Day, and will include their popular bran pies, Christmas fare, and a board-room concert in the evening.

### PRINCESS LOUISE AND JUBILEE NURSES.

Last week Princess Louise visited the Training Home of the Scottish branch of Queen Victoria's Jubilee Institute for Nurses at Edinburgh, in order to present the nurses with their badges of proficiency, and with certificates marking a period of service. Her Royal Highness, in the presence of a large gathering of nurses, personally handed badges and brassards to 19 young women who have qualified for employment as Queen's nurses, and certificates to 15 who have had two years' experience in that capacity. Seven badges and brassards, and 12 certificates were granted in absence. A vote of thanks to the Princess for her attendance and performance of the ceremony having been carried with enthusiasm, the Duke of Argyll, replying on her behalf, said that she had found great pleasure in being present, and in meeting nurses from all parts of Scotland. He thought that "there must have been some conspiracy among them to get their patients well for that particular occasion in order that the nurses might attend in such force;" and he asked them to tell the nurses who were on duty that the Princess regretted that she was unable to visit them this year, and hoped that it would be their turn to come next season and receive their badges from her. The Princess was subsequently conducted over the building by the superintendent, Miss Wade.

### LADY DUDLEY'S APPEAL.

Simultaneously with the appeal of the Countess of Dudley on behalf of the sick poor in Ireland, we have received a copy of the fourth annual report of the West of Ireland Association at Manchester, by whose aid two district nurses in Oughterard, Galway, and Ballintubber, Mayo, are maintained. In this report it is mentioned that the whole community is under a deep obligation to her Excellency for the stimulus of her example. Lady Dudley, in support of her appeal to the English people, proves by the citation of a typical case the hopelessness of expecting certain districts to provide a nurse at the expense of the rates. In the Western Union of Knocknalower, which she has herself visited, there are only, in an area of 70,000 acres, 5,000 wretched cabins of small landowners, and so poverty-stricken is the place that no fewer than ten doctors have resigned during the past five years. The valuation is only 12s. 2d., while the rates amount to 5s. 10d. on houses and 2s. 9d. on lands. In many parts the people have to journey a whole day across the bogs in order to obtain medical relief. In such circumstances, the presence of a qualified district nurse would indeed be a boon; and we do not think that a deaf ear will be turned at this season of the year to Lady Dudley's plea for the help of the charitable.

### THE CORONATION NURSING FUND.

At the first annual meeting of subscribers to King Edward VII. Coronation National Fund for Nurses in Ireland, on Monday, it was announced by the chairman that since November 30 over 60 new members, all nurses, had joined the movement. The receipts for the year were, he stated, £3,865 and the disbursement £494. On the motion for the election of the Subscribers' Committee a number of persons present protested that the business element was not sufficiently represented on the list suggested, and proposed the adjournment of the meeting. Eventually this proposal was relinquished, and the names were accepted. The following nurses' representatives were elected on the Council of the Fund: Miss Kelly, Stevens' Hospital; Miss M'Donnell, Richmond Hospital; Miss Humpson, Portobello; Miss Shooter, City of Dublin Hospital; and Miss Lamont, superintendent of the Irish Branch of the Jubilee Nurses' Institute.

### THE RESIGNATIONS AT CHARING CROSS HOSPITAL.

The Council of Charing Cross Hospital, we are informed, accepted at their ordinary meeting last week the resignations of the three sisters who were invited to tender them, and passed a vote of thanks to them for their past services. The resignations of four other sisters were also accepted.

### GLASGOW AND WEST OF SCOTLAND CO-OPERATION OF TRAINED NURSES.

The eleventh annual report of the Glasgow and West of Scotland Co-operation of Trained Nurses, whose annual meeting is being held this week, shows that the number of nurses on the roll at the end of October, 1903, is 160, two less than last year; and the number of cases nursed 1,629, upwards of a hundred fewer. The earnings, on the other hand, were £10,046, or more than £400 in excess of 1902. It is stated in the report that the committee have had "under consideration the question of sick and pension funds for nurses."

### NURSES AND HOSPITAL CONSTUCTION.

In an interview with our Commissioner, which we report this week, the Matron of Darlington Hospital, dwelt on the importance of nurses acquiring a knowledge of hospital construction. Her own staff have had the advantage of having the construction of the building, which is now in process of extension, being practically explained to them as it went along, and it is satisfactory to learn that they have taken an interest in the work. We entirely share the view that those who enter hospitals, with the idea of spending a considerable portion of their lives in them, should learn to understand the principles of construction. The knowledge will always prove useful; it may at any time become essential.

### ALLEGED SCANDAL AT HACKNEY WORKHOUSE INFIRMARY.

The Local Government Board have been asked by the Hackney Board of Guardians to conduct an official inquiry into some serious charges made by nurses in Hackney Workhouse Infirmary against a member of the board. The guardian in question is said to have entered a lavatory, and when told by a nurse that it was private, to have used insulting language both to her and to other nurses who witnessed the incident. A visitor to one of the wards has stated that he heard the guardian's language and told him that he was not a gentleman, whereupon the guardian struck him. The guardian declares that he had intended to examine the lift, not the lavatory, when the nurse requested him to leave the ward, and he suggests that there is a conspiracy against him.

### AN INJUSTICE TO MIDWIVES TRAINED IN IRELAND.

The regulations of the Central Midwives Board, under the Act of 1902, shut out from admission to the roll the midwives who have been trained in Irish hospitals. Dr. Alfred J. Smith, gynæcologist to St. Vincent's Hospital, Dublin, cites two provisions in the first clause regulating the course of training which, he affirms, have this effect. The first is that a person presenting herself for examination must have attended and personally delivered twenty cases of labour, and the second that she must have nursed twenty cases for ten days after confinement. It is impossible, Dr. Smith states, for hospitals such as the Rotunda, the Coombe, and the National, to give twenty cases to a nurse for personal conduction, because to do so would necessitate doubling the size of the hospital; and it is equally impossible to enable a woman to nurse the patient for ten days following labour, because the patient leaves the hospital on the eighth day, and would not remain longer even if the hospital authorities could keep her. Without endorsing the opinion of Dr. Alfred Smith that midwives trained for six months in Irish midwifery hospitals "are far superior to the average woman who will present herself for examination after having fulfilled the three months' midwifery tutelage required by the Central Midwives Board," we certainly agree with him that midwives who have obtained their certificate from the three excellent schools he names should be entitled to be admitted to the examination of the Central Midwives Board without having to spend a further three months in England. If a condition of affairs which is obviously calculated to seriously injure Irish training institutions of acknowledged merit cannot be rectified by any other means save the amendment of the Act itself, then the Act must be amended.

### RESCUE OF A NURSE BY A MATRON AT HULL.

It is the admirable custom of Miss Dunn, the lady superintendent of the Hull Jubilee District Nursing Association, to periodically accompany each nurse round her district. The other morning the nurse whose patients she proposed to visit was knocked down by a horse and waggon, and but for the intrepidity and presence of mind of the matron, she would have been crushed to death by the wheels of the cart. Miss Dunn, however, who, except a few children, was the only person on the spot, rushed to the horse's head and managed to push the animal on one side, thus enabling the nurse to get from under his feet. The nurse, happily, was unhurt, but Miss

Dunn kindly proposed to send her back to the home and take the visits herself. We agree with the correspondent who sends us this information, unknown to Miss Dunn, that such brave conduct ought not to pass unnoticed.

### NURSES AND NERVE TRAINING.

Nerve Training in Relation to Physical Culture was the subject of an interesting lecture last week to the nurses of Chelsea Infirmary, by Mrs. William Archer. The interchange of activity and rest constituted, the lecturer said, the rhythm of life; many people, however, lost sight of the necessity for rest, and what followed was called over-strain, or, in its extreme form, mental breakdown. Active people were apt to waste an amount of nervous energy which ought to be stored up for work; thus, a woman would dash into a cab in order to catch a train, and then, instead of sitting calmly and leaving horse and driver to do the hurrying for her, would expend her energy in needless clutching of umbrella, luggage, etc., and would go through all the agony of losing her train beforehand, whether she eventually caught it or not. This was waste of force, and it was worry, not work, that caused over-strain. The over-strained woman did not rest even at night, but was tense, often sleepless, and busy evolving plans for fresh activities on the morrow. She implored the nurses not to hound themselves into collapse while they were in their best and most vigorous years. They should learn to drop tension whenever possible; to abstain from superfluous tension, and to apply tension proportionately. A great many questions were asked at the conclusion of the lecture, and a demonstration was given by Mrs. Archer, who showed how to relax tension completely, and recommended the nurses to spend 20 minutes daily in absolute rest on the floor, the body and limbs being entirely passive and deep breaths being taken at intervals of two minutes.

### AMERICAN INDISCRETION.

The fact that several hundred American women have offered their services as nurses to Japan in the event of war with Russia is cited as evidence of their alertness. It is certainly evidence of their indiscretion. Unless or until hostilities between Japan and Russia are announced most people in the civilised world will hope that war will not take place. Moreover, the offer by the American nurses of their services to Japan, however well intended, is distinctly calculated to embarrass the Government of the United States. Naturally the Japanese Minister to whom the offer was made expressed himself impressed with it, but it was as injudicious as it was premature.

### A TRAINED NURSE FOR GRANARD HOSPITAL.

It may be hoped that we have, for a time at any rate, heard the last of the dispute at Granard Union Hospital. The Bishop of Ardagh, in declaring himself unable to accede to the request that the nuns should resume nursing in the Granard Union Hospital, strongly appealed to every member of the Board of Guardians "to bring to an immediate end this weary, long-standing contention." The appeal was not made in vain, for at the last meeting of the board, the chairman suggested that it should be decided to appoint a trained nurse, and after some discussion the proposal was agreed to. The nurse selected may not find her path strewn with roses, but if she combines tact with knowledge, she may prevent another outburst of the waters of strife.

### DUBLIN NURSES AND THEIR PHYSICIAN.

Dr. Harley, who for nearly 20 years has been a director of the City of Dublin Nursing Institution and is physician to the nursing staff, has been presented with an address and some silver plate by the nurses. Mrs. Butler, one of the staff, who designed the address, made the presentation to Dr. Harley on behalf of the nurses of the institution past and present, the recipient acknowledging it in suitable terms.

### EXAMINATION AT BRISTOL GENERAL HOSPITAL.

The following is the result of the examination at Bristol General Hospital:—Second Year Nurses.—Anatomy: 1st prize, Nurse Wood; 2nd prize, Nurse Beattie. Medical Nursing: 1st prize, Nurse Just; 2nd prize, Nurse Lena Davies. Surgery: 2nd prize, Nurse Beattie. Physiology: 1st prize, Nurse Lane; 2nd prize, Nurse Wood. First Year Nurses.—Anatomy: 1st prize, Nurse Skinner; 2nd prize, Nurse Coomber. Medical Nursing: 1st prize, Nurse Philo; 2nd prize, Nurse Coles. Physiology: 1st prize, Nurse Coomber; 2nd prize, Nurse Jenkyns. Surgery: 1st prize, Nurse Coomber; 2nd prize, Nurse Britt. Gold Medal: Nurse Just. Silver Medal: Nurse Fanny Nicholls. Certificate of Merit: Nurses Bibbing, Grace Davies, Bowyer, Grant, Hawkins, and White. The prizes and certificates were distributed by the chairman, Mr. J. Storrs Fry.

### EXAMINATION AT KINGSTON INFIRMARY.

Mr. James Cantlie, F.R.C.S., reports that he has submitted Nurses Florence Gibbs, J. Holland, and Ellen Louise Morley, of Kingston Infirmary, to a written and viva-voce examination, and found all three to be worthy of certificates of proficiency. He adds that the knowledge of anatomy and physiology they displayed was excellent, and much above the average; that the subjects of ventilation, ward accommodation, and hospital hygiene have been well taught; and that practical nursing and the personal care of the sick have been thoroughly mastered by them.

### SHORT ITEMS.

Miss Margaret Steenson has been appointed, provisionally, sister in Queen Alexandra's Imperial Military Nursing Service, and Miss A. F. Fitzgerald has been appointed staff nurse.—On the recommendation of the Stockport Guardians, the Local Government Board have sanctioned the increase of the salary of Miss Hall, matron of the Stockport Union Infirmary, who also undertakes the duties of superintendent nurse, from £100 to £110.—The bazaar at Brighton last month has resulted in the handsome sum of £637 being handed over to the Brighton, Hove, and Preston District Nursing Association.—Miss Bulkeley Williams, who has just been appointed lady health visitor for Acton under the Urban District Council, received her training from the National Health Society.—Miss J. Lumsdaine, who has been matron in the Home for Private Patients at Montrose Royal Asylum for the last 26 years, has retired from active work and has been granted a pension of £50 per annum.

### Lectures Upon the Nursing of Mental Diseases.

By Robert Jones, M.D.Lond., B.S., F.R.C.S.Eng., M.R.C.P.Lond., Resident Physician and Superintendent of the London County Asylum, Claybury.

### LECTURE II.

The brain of man is the organ of his mind. How mind and matter are inter-related is not at all clear, and it is customary to describe the relationship as a parallelism—material action on the one hand and mental action on the other. The brain does not secrete mind as the liver does bile; and mind, so far as we know, is non-existent without matter. The spirit of life, or consciousness, depends upon a proper flow of healthy blood into the brain. If this be interfered with—as for instance by pressure upon the two carotid arteries, which suddenly diminishes the blood supply to the brain—unconsciousness may result. When a person faints through loss of tone in the blood vessels, or from sudden failure of the heart unconsciousness results from an interference with the blood supply. The same may result from a violent blow on the head, which brings about a disarrangement of the delicate structure of the brain. Certain drugs, as opium, chloroform, alcohol, etc., may alter the quality of the blood circulating in the brain and produce unconsciousness. Disorder of the brain through disease may in diverse ways alter consciousness, so that through disease the mind may be permanently impaired or lost. From a variety of causes, therefore, it is abundantly clear that consciousness, or mind, depends upon a normal condition of the cortex of the brain, and that a man loses consciousness so soon as this part of his cerebral hemispheres ceases to act. The science of the healthy mind is technically termed Psychology, and some elementary acquaintance with this science is necessary before we can consider abnormal conditions.

Psychology, within very recent times, has made great progress, for it is now considered to be the study of mind in its widest possible range, including the minds of animals from the very beginnings of life (comparative psychology), up through the mind of children (genetic psychology) to that of adult men and women. Of the conclusions found, much has been obtained by experimental and highly scientific investigation (experimental or physiological psychology). At one time the only method of investigation in vogue was one which is possible to every person, viz., introspection or self-examination—noting one's own mind, and watching the changes in the emotions, memories, and associations as circumstances occurred from time to time—the only method of examining the mind directly.

Further information in regard to the mind is obtained through the subject of our Lectures, viz., mental diseases—a branch of psychology which has been referred to as abnormal psychology or mental pathology.

Apart from certain inherited tendencies termed "instincts," and which will be referred to later, it may be definitely stated that our mind depends upon our experience, or what comes into the mind through the avenues of the senses; not only of the five special ones, but also through the muscular and the organic senses connected with the inner vital organs, through the senses of heat and cold, which are probably distinct from that of touch, as also through many others possibly having some special apparatus, such as the joints, giving sensations of pressure and of the equilibrium or proper balancing of the body. It is quite clear that our beliefs, knowledge, and opinions, must be based, and be dependent, upon the supply of material from the senses; the mind makes up its thoughts and re-acts from the supply brought to it through the senses. It is the tendency of every sensation that comes into the mind to pass out into action and this is very important. Persons born blind cannot have delusions of sight, nor can congenital deaf mutes have aural hallucinations. This reaction of the mind to stimulus is capable of control by a higher power called "inhibition" which in the main is the result of education. It is this higher controlling power, the power to say "no," which differentiates man from the lower animals as well as the different races and divisions of men from each other. This inhibition is the basis of deliberation, such as, when one sensation or idea is stopped by another—a man crosses the road, but a coming danger "inhibits" this movement. He first hesitates, then deliberates and finally desists. The "holding" process by which the mind retains the material it receives, is through the "memory," a peculiar feature of nerve cells which we may suppose to be a molecular change in the cells persisting for a longer or shorter time according to the intensity of the particular experience. A further process is "association of ideas" whereby those things which have been perceived or recognised through the senses (Perception), tend to come up again in the future in the same combination, and based upon which process the different ideas are combined. For example our mind associates certain clothes with certain days, such as Sunday suits, certain emblems with certain seasons, holly leaves and berries with Christmas, certain flowers with certain persons who wear them, and so on. The comparing of new materials with past experience is completed through "assimilation," such as when a scene on the stage recalls a familiar landscape, but the combining tendency of the mind, by means of which things that are grouped together through the "association of ideas" come before the mind is called "Apperception." This is a word over which much has been said and written, but it only means that special and peculiar activity of the mind which combines all it receives into larger and more definite combinations and higher degrees of perplexity and to which combinations the name, notion or concept, or general idea is applied. "Association of ideas" refers to the mental materials which tend to come up together and not to the mind's activity. "Apperception" refers to the mind's activity. The "whole" mind acts in the way we describe. This state of the mind is called the stream of consciousness. This stream always flows on and there are no breaks. Sensations from the vital organs, muscles, sense organs, are always flowing into the mind, giving rise to either comfort or discomfort. This feeling may be associated with some idea or thought in the mind, which is the origin of the "emotions," and we may then be filled with hope, fear, love or hate as the case may be. It is this association of a sensation with a thought which constitutes what we call an "emotion." As regards the emotions, the feeling of pain or pleasure enters largely into the attitude which the mind takes in regard to its experience or action. To understand this in others we go back to introspection. We infer this or that thought from this or that action because we know from experience in ourselves that when we act in a particular way certain thoughts or feelings inevitably accompany or precede these actions. Our actions, therefore, are the result of our thoughts, so that whatever our thoughts may be, these thoughts tend to arouse in us their corresponding train of suggested actions. These actions may be brought about by the influence of our perceptions, or the memory of these perceptions, or through the exercise of the imagination—taking these imaginations to be realities. Sometimes things are presented to the mind from within, as in dreams, or in a fantastic manner from without, yet in each case so apparently real as to deceive the mind; when this is the case, and the presentation is untrue, but still believed in, it is an illusion or a hallucination. An illusion points to an error in "assimilation," the mind is unable to compare correctly a past experience with the present, and an impression appears in consciousness very different from the actual fact. Hallucinations arise when no impression is produced upon any of the senses. They are false perceptions, arising from within and are quite independent of outward objects. If the tolling of a bell be heard and be interpreted as the sound of a human voice, it is an illusion; but when no sound is audible, and human voices are declared to be heard, it is a hallucination. Delusions, on the other hand, pertain to the mind only, and not to the senses. Such a condition is due to some perversion; what, is not always known—which prevents the subject from realising the falsity of his belief, by the evidence of his own senses. A person calling himself the Saviour, or the Creator, or stating he is 10,000 years old, labours under a delusion.

### District Nursing and Diet.

### EXAMINATION QUESTIONS FOR NURSES.

The question was as follows:—If called upon in district nursing or otherwise to arrange the diet of a person suffering from an exhausting disease such as consumption, and if that person belonged to a labourer's family whose wages did not exceed 18s. or thereabouts per week, what should you advise as the cheapest and most nourishing scale of diet?

### General Result of Competition Good.

This month the papers are very good, taken as a whole, and show considerable thought, but I shall give the question again later on, and hope to have still better results. One of the chief defects is that the candidates make the mistake of occupying themselves with apportioning the meagre 18s. weekly.

This is unnecessary as in practical experience they would not be allowed to know anything of the general expenditure, the poor usually jealously guard their domestic affairs from the general eye. The idea in giving this question was that it should be a test of a nurse's knowledge of the different values of food obtainable, and this result has hardly been reached. For this reason it will be well that our competitors should think the matter over and try again next year. Another fault is the result of an inadequate knowledge of the narrow proportions of 18s. weekly. Almost all make the same mistake, even the winner of the first prize. In no family, however small, could 6s., or even 5s. be devoted entirely to one person.

### The Prize Winners.

"Aubrey" carries off the first on account of his generally good answer, but he would have been displaced by "Sister N." if the latter had not erred in writing her paper in divisions and under headings. "Aubrey" seems to understand advising the housewife, and evidently would make no mean one himself. He is quite right and unappetising as the idea of the discoloured meat is, there is no harm in it, and it falls wonderfully in price. One thing which he notes, and which is all important, is the giving of dripping in place of butter; it is more economical and extremely nourishing, and administered in the form of fried bread it makes a good meal. "Sister N." notes the value of cheese. "Plodder" gains the second prize because she suggests a great variety of cheap foods that are at the same time useful.

### First Prize.

The only way an invalid's life could be made bearable under the circumstances named would be to practise strict economy in everything, more especially the purchase of food. I should advise the thrifty housewife to get the meat for the week late on Saturday night just as the shops are shutting; when for a small sum and with a little haggling sufficient meat can be procured to last a week. When meat has been hanging in a butcher's shop all day and gets discoloured by the glare of his gas, it sells for an incredibly low price. The fat parts of the joint must be cut off and melted down for dripping to eke out the scanty allowance of butter which the family can barely afford. Vegetables should be bought in the same manner by visiting the market when the dealers are closing. Bread and milk do not vary much in price and foreign eggs are cheap and fairly good. In the average labourer's family there are several children to provide for out of the weekly wages, and with rent and other expenses only about 5s. or 6s. could be spared for the patient. Every scrap of food must be carefully portioned out to the members of the family as only an experienced house wife knows how.

I should advise that the patient has three good meals a day and not less than two pints of milk. Porridge and milky tea for breakfast, meat and some simple pudding as rice or tapioca for dinner, bread and butter and warm milk for tea, with more milk at about eight in the evening. Toast, poached egg, and fried bread make pleasant and cheap varieties.

It is better to give the patient a small quantity of food at one time than it is to give him a lot and waste what he leaves. If he suffers much with thirst a cheap drink may be made by cutting a lemon up into slices and pouring boiling water on it. "Aubrey."

### Second Prize.

Diet for patient of the labouring class suffering from exhausting disease such as phthisis.

If in a country district dairy produce—milk, eggs, butter, cream—would be cheaper and of better quality than in London, also vegetables. I should suggest the following:—

*Breakfast.*—Oatmeal porridge, hot milk, brown bread and butter with an egg if they were plentiful, say from 16 to 20 for 1s.

*Lunch.*—Egg and milk, cocoa made with milk, plain boiled milk.

*Dinner.*—Mutton (inexpensive part of neck) stewed with barley, tripe, cow-heel, or calves-foot, fat bacon, rabbit, broth made from sheep's head, or from the inexpensive part of shin of beef, thickened with rice, barley, lentils, peaflour. Vegetables, such as potatoes, parsnips, carrots, lentils, turnips, artichokes, onions, cabbages, etc. Light pudding, rice, sago, tapioca, custard, accompanied by stewed fruit if cheap and plentiful, junket.

*Tea.*—Coffee, tea, or cocoa made with milk, buttered toast, or bread with plenty of butter. Eggs poached, buttered, or lightly boiled.

*Supper.*—Bread and milk, porridge, onions cooked with milk and butter, broth gruel made with milk, egg-flip.

At least one pint of milk should be given between the supper and breakfast meals, say half a-pint last thing at night and same quantity in the early morning. All milk should be sterilised. If plain milk disagreed it might be diluted with barley-water, and the latter flavoured with lemon-juice makes a refreshing drink.

If in London, or a town where fish was cheap and plentiful, the inexpensive varieties such as hake, eels, cod, whiting, plaice, fresh haddock might be added to the dietary.

Cream during the summer months would be procurable in the country. "Plodder."

### Honourable Mention.

This is gained by "Sister N.", "Nurse F.", "Damaris," and "R. I. R.", a male nurse. In considering the question another time remember that cream and such delicacies are out of the question.

### Answers by Post Impossible.

All successful candidates will in due course receive notice, and no correspondence can be undertaken. If two choose the same pseudomyn it is of no conseqnence; the fortunate one will be informed.

Question for December.

If ordered to apply constant hot irrigation to a leg with a compound fracture in a cottage where appliances were limited, how would you proceed?

The Examiner.

Rules.

The competition is open to all. Answers must not exceed 500 words, and must be written on one side of the paper only. The pseudonym, as well as the proper name and address, must be written on the *same* paper, and *not on a separate sheet.* Papers may be sent in for fifteen days only from the day of the publication of the question. All illustrations strictly prohibited. Failure to comply with these rules will disqualify the candidate for competition. Prizes will be awarded for the two best answers. Papers to be sent to "The Editor," with "Examination" written on the left-hand corner of the envelope.

N.B.—The decision of the examiner is final, and no correspondence on the subject can be entertained.

In addition to two prizes honourable mention cards will be awarded to those who have sent in exceptionally good papers.

### The Nurses of Darlington Hospital.

### A CHAT WITH THE LADY SUPERINTENDENT: BY OUR COMMISSIONER.

When I visited Darlington Hospital at the end of last month the lady superintendent expressed her regret that I should have come at a time when confusion, caused by extensions which are being made in every department, prevailed. But I assured her that from my point of view I could not have done better, since I had an opportunity of seeing how the ordinary work was being pursued unaffected by the din and dust of building operations. I was also able to form an excellent idea of what the hospital will be like when the extension is completed, thanks to the courtesy of Miss Hunt under whose auspices I made an inspection of both the original and the new portions. As we proceeded, she explained the scheme of extension which includes two additional wards of 14 beds each, two rooms for a single patient, a laundry of considerable size, and a mortuary. There is an admirable arrangement with regard to the latter, in the shape of a room, not connected with the mortuary, from which people can view the bodies. The extension also embraces a new operating-room and everything complete in the shape of fittings, a new sitting-room in the nurses' quarters, several bedrooms, a dining-room near the kitchen, and a new housemaid's pantry and larder. When these are finished the hospital will not be unworthy of a growing and important town like Darlington. This, however, is not the first extension, for as the lady superintendent said:

"When I came here seventeen years ago with the idea of staying six months, the hospital was a comparatively new building, and since then we have added servants' quarters, for which a sum of £550 was given to us by the Misses Pease, of Southend; the porter's lodge, containing four rooms; and the children's ward, the building of which was made possible by a bequest from the late Mr. Cox, who gave £2,500 towards it, and £2,500 for the proper endowment. Miss Emma Pease, one of the donors of the £550 for the servants' rooms—who formerly had a small children's hospital of her own in the town which she handed over to us —also contributed substantially to the work."

### The Necessity for Extension.

"How many beds are there in the children's ward?"

"Fourteen, and 26 in the two wards for adults. But though our accommodation is only for 40 patients, we have lately been obliged to put 55 into the wards. Altogether the position was very grave, and the committee recognising the evils of overcrowding, and realising the strain of the anxiety caused to the staff, made up their minds to appeal for £12,000 in order to obtain adequate provision for the growing needs of the town."

"How have the residents responded to the appeal?"

"Fairly, but there is still a balance of £5,500 due. We think that if the honorary surgeons are willing to give their time and skill for the benefit of the patients, the town, which is very prosperous, should be prepared to provide the building and appliances which are necessary for an admittedly excellent work. Of the money already subscribed £1,000 was given by a lady who desired that the hospital should possess an up-to-date operating room, and £1,000 by one of the gentlemen belonging to the Committee."

"When did you begin the extension?"

"In January, and in spite of the discomfort to everyone during the process of building, we have taken in more patients than ever during this year. This emphasises in a striking manner the urgent need of extension."

"Have you a large percentage of serious cases?"

"The number of operations and serious cases has steadily increased. Notwithstanding the drawbacks of the existing building, 355 operations have been performed, many of them of a most critical nature since its opening."

"Do the working men show their practical appreciation of the advantages they derive from the institution?"

"They subscribe admirably, and have been making considerable efforts towards the extension fund. Many of them contribute a penny or twopence a week to the support of the hospital, and others have helped by giving a day's work, a very useful way of showing their desire to assist."

### Surgical Cases.

"With regard to the work," continued the lady superintendent, "it has necessarily been limited, almost entirely, to surgical cases. Indeed, that is one of the reasons that we want the new wards, which we hope will enable us to receive more medical cases.

"You might give me an idea of a few of the cases."

"We have a large number of operations for tuberculous disease of bones and glands, for varicose veins, hernia, etc. A considerable number of eye operations are done by the ophthalmic surgeon. Although there are some very large works in the town I am thankful to say that amputations are infrequent. There have been between 40 and 50 abdominal sections this year, including enterectomy, gastro-enterostomy, choledochotomy, operations for perforated gastric ulcer, appendicitis, and the like. We believe that our results have been quite as good as those of the large hospitals."

### The Training School.

"How large is your nursing staff?"

"There is a staff sister who is responsible for the work throughout the hospital, and she attends all the operations; a night nurse, five of our own probationers, and two probationers who are being trained for the North Riding Rural Nursing Association."

"What is your period of training?"

"The probationers enter for two years, with the possibility of remaining three if arrangements can be made. Some have already commenced their third year, and the enlargement of the hospital, will, I hope, hasten the lengthening of the period to a fixed time of three years. A probationer in her third year assists in the children's ward, which is entirely separate from the other wards of the hospital. With her are two Norland nurses and a night nurse.

NORLAND NURSES.

"How long have you had Norland nurses?"

"For some years. They come for three months, have the work of the wards to do, receive instruction from the staff sister, and are allowed to attend the lectures.

"Under what conditions do they come?"

"The Norland Institute pays at the rate of 10s. a week, so that we get £52 a year towards maintenance. We generally find them very intelligent and capable."

"When did you start the training school?"

"Between four and five years ago. Our own probationers receive £8 the first year, £12 the second, and £18 the third, with indoor uniform. The pupils of the North Riding Association come for a year and pay a fee."

AGE AND HOURS OF DUTY.

"Of course you have an age limit?"

"We do not like to take probationers who are under 24. With regard to uniform, I should like to say that I prefer it not to be worn outside the hospital. If a nurse goes about the town in her uniform she is known by everybody, and people stop and ask her about the patients, which is not at all desirable."

"What time off duty do the nurses get?"

"Two hours every day and half a day once a month. They go into the wards at 7 A.M. and leave at 8.30 P.M. The probationers have a fortnight's holiday and the staff nurses three weeks."

"I suppose they receive a course of theoretical instruction?"

"An exhaustive series of lectures has been given during the last eighteen months by the house surgeon, and I have a class weekly.

"Were you at the London before you came here?"

"No. I was trained at St. Thomas's Hospital, and the only appointment I held before this was that of matron and secretary of the new Hospital for Women, Marylebone Road."

HOSPITAL CONSTRUCTION.

"There is one drawback I noticed in your nurses' quarters. They do not all have a separate bedroom."

"No. Unfortunately when the home was built, it had to be arranged over existing buildings, consisting partly of the out-patients' department, which is here one of great importance, the nurses taking turns in attending and assisting the house surgeon in dressing the wounds. There are several, as you see, who have separate rooms, and I hope later on every nurse will have one to herself. We have been so pressed for space lately, that nurses have been obliged to sleep in the Committee Room. They have gone through a period of great discomfort with little complaining, but they have also had the advantage of having the construction of the building practically explained to them as it went along."

"Have they taken an interest in construction?"

"Yes, I am glad to say they have. One scarcely realises how important it is for those who enter hospitals to understand the principles of construction, and to know when they see a plan what it is likely to result in when it is completed. In my opinion it is most essential for the matron or lady superintendent of a hospital, and therefore for all nurses, to acquire a knowledge of hospital construction."

Subsequently Miss Hunt informed me that the Committee of the Hospital consists of twenty persons of each sex—that some of the members are working men, and that all of them take a great interest in the institution. Certainly the appearance of the old wards, well-ordered, bright, and adequately equipped, reflect credit on the management, and warrant the expectation that when the extension is finished, the inhabitants of Darlington will consider it a pleasure, as well as a duty, to make haste to relieve it of debt.

### Appointments.

BRISTOL GENERAL HOSPITAL.—Miss C. C. Edwards has been appointed home sister. She was trained at the Bristol General Hospital.

CAMBERWELL INFIRMARY.—Miss Alice L. Harris has been appointed sister. She was trained at Middlesex Hospital, where she was afterwards staff nurse. She has also been staff nurse at Addenbrooke's Hospital, Cambridge. She holds the L.O.S. certificate.

COLCHESTER HOSPITAL.—Miss M. Dietz has been appointed day sister, and Miss Ada Morgan night sister. Miss Dietz was trained at Radcliffe Infirmary, Oxford. Miss Morgan was trained at Colchester Hospital. She has since been staff nurse at the National Hospital, Queen Square, Bloomsbury, sister at Stockwell Fever Hospital, and district nurse in connection with the Cambridge University Union, Camberwell.

MONTROSE ROYAL ASYLUM.—Miss Julia Gass has been appointed matron in the Home for Private Patients. She was trained at the Edinburgh Royal Infirmary, where she was for some time staff nurse, and subsequently she was sister superintendent at the Kasr-el-Aini Hospital, Cairo. For the last 12 months she has been assistant matron at Carnegie House.

NEW ORPHAN HOUSES, ASHBY DAM, BRISTOL.—Miss A. Bergin has been appointed superintendent nurse. She was trained at Bristol General Hospital, and has been casualty sister in the same institution for the past four years.

ROYAL CHILDREN'S HOSPITAL, BRISTOL.—Miss White has been appointed sister. She was trained at Bristol Genera Hospital.

ROYAL HOSPITAL FOR SICK CHILDREN, ABERDEEN—Miss Annie A. Smith has been appointed sister. She was trained at the Poplar and Stepney Sick Asylum, where she was afterwards staff nurse and sister. She has since been charge nurse at the Western Hospital, Fulham, S.W.

ST. LEONARD'S INFIRMARY, SHOREDITCH.—Miss Florence Farrow has been appointed night sister. She was trained at Huddersfield Infirmary, and has since been charge nurse at the Seamen's Hospital, Ramsgate, and charge nurse at the Western Hospital, Fulham, S.W.

ST. PANCRAS INFIRMARY, COOK'S TERRACE, N.W.—Miss M. Thomson has been appointed assistant matron. She was trained at St. Saviour's Infirmary, East Dulwich, has been ward sister at the Poplar and Stepney Sick Asylum, nurse at the British Hospital, Algiers, ward sister and night sister at St. Pancras Infirmary, Cook's Terrace.

SALFORD UNION INFIRMARY.—Miss Mary Watson Wayne has been appointed assistant matron. She was trained at the London Hospital, Whitechapel, where she afterwards held the post of staff nurse. She has since been sister of the women's medical ward at the Taunton and Somerset Hospital, and sister of the male operation ward at the North Staffordshire Infirmary.

SHARDLOW JOINT HOSPITAL, DRAYCOTT. — Miss Ida Maud Moss has been appointed superintendent nurse. She was trained at Farnworth Hospital and the City Infirmary, Birmingham. She has since been sister at Brook Fever Hospital, London; matron of the Stone Isolation Hospital, and matron of the new Isolation Hospital, Pontardawe.

WINCHFIELD UNION INFIRMARY.—Miss Margaret Fisher has been appointed assistant nurse. She was trained for three years at St. Peter's Home, Woking, by the Meath Workhouse Nursing Association.

WOLVERHAMPTON AND STAFFORDSHIRE GENERAL HOSPITAL.—Miss Caroline Turner has been appointed sister. She was trained at the Liverpool Royal Infirmary.

### Christmas Books.

*Concluded from page* 154.

G. Newnes, Limited.

"The Work of Botticelli" (3s. 6d. net). This artistically got up volume contains an interesting introduction by Richard Davey and 64 full-page illustrations, beautifully reproduced, from the artist's best-known pictures. It would make a charming Christmas gift-book. One of Newnes's "Art Library" Series.

"Richard Savage." By Charles Whitehead (3s. net). This is a new volume from Newnes's "Thin-paper reprints of famous novels." It has a charming cover, limp green leather with a decorative design in gold. The paper is perfectly opaque and the book light to hold. The original edition of "Richard Savage" appeared in 1842.

"The Adventures of Gerard." By Conan Doyle (6s.). These adventures are told in Sir Conan Doyle's best manner in a series of tales which have to do with Napoleonic soldiers and events in an age which, says the author "was rich in military material, most human, and most picturesque." Just the book for boys who like historic fiction, based on fact. It is profusely illustrated.

The Walter Scott Publishing Company, Limited.

"Barty's Star." By Norman Gale (2s. 6d.). This book is perhaps best described as the story of a child for grown up people, and as such with its charming illu strations, will give pleasure to the numerous persons for whom such a class of literature has a fascination.

"Dramatic Sonnets, Poems and Ballads." From the Poems of Eugene Lee Hamilton (1s.). This latest edition to the "Canterbury Poets" contains some beautiful sonnets and short poems. A scholarly introduction contributed by Mr. W. Sharp adds to the interest of the little book, as it gives a brief biography of the author, and the circumstances under which much of the best verse was written.

Chatto and Windus.

"In Lakeland, Dells and Fells." By W. T. Palmer (6s.), Mr. Palmer's new book forms a pleasing complement to his previous one, "Lake Country Rambles." Written with the eye of a lover of nature, a sportsman and a keen observer, it is refreshing to read, and brings a breath of the fells with it. It is just the book for a man.

"A Butterfly: her Friends and her Fortunes," by Iza Duffus Hardy (6s.). The heroine of this novel gives up a young and impecunious lover, as her family appear to wish it, and marries a "sturdy Republican," Hiram Holdsworth, who is a South American millionaire. She returns with him to his estate in Florida, where she is joined two years later by a friend, Janet Drummond. Then the sturdy Republican sets to work in a business-like way to make advances to her friend. Failing to make any impression, except one of utter disgust, he entraps her on board his yacht under the promise that she will meet there a party of friends, her fiancé, Roland Clavering, among them. Some exciting chapters follow. Janet is finally united to Roland, whilst Hiram Holdsworth dies from shock, and his little wife, after a discreet interval, marries her first lover.

"Stepping Blindfold," by I. W. Spright (3s. 6d.). In "Stepping Blindfold" the reader follows the career of Roden Joslyn, an unprincipled youth, and the events which occur during a holiday spent in Normandy.

"Prince Hagen," by Upton Sinclair (3s. 6d.). This book, described by the author as "a phantasy," has to do with Prince Hagen, the son of the Nibelungen Duke Alberich, who murdered Siegfried, the hero of Wagner's opera, "The Nibelung Ring." He is made to appear on earth, by the author, and is favourably received into the inner circle of American society.

BOUND MAGAZINES AND MISCELLANEOUS BOOKS.

G. Newnes, Limited:—"The Wide World Magazine," vol. xi., is as usual a storehouse of exciting narratives of sport and adventures in many lands. Price 6s. 6d. "The Captain," vol. ix., May to September, 1903 (price 6s.), has everything in it that boys delight in. The illustrations and stories are alike excellent, and the present volume upholds the standard of this popular magazine. "Peccavi," by E. W. Hornung, and "Memoirs of a Mother-in-Law," are two novels issued by Newnes in their sixpenny library (6d. each). Methuen and Co.:—"A Book of Bad Children" is very funny, with its queer little pictures, and quaint rhymes (2s. 6d.). "Larks and Levites," a pack of nonsense, by Leonard Larkin, comes from the same publishers (1s.). "England Day by Day." By the authors of "Wisdom While You Wait." (Methuen and Co. 1s. net.) This witty shillingsworth is a book to have at hand, for it is full of practical suggestions. "The Prophetic Calendar" will be invaluable to those who wish to be "up to date" in the events of 1904. "Little Folks," (Cassell and Co., 3s. 6d.), is as attractive as ever. "Little Folks Song Book" (2s. 6d.), with four coloured plates, is a bright collection of songs for children, with simple accompaniments to pretty tunes and pretty words. "The Child Wonderful" (2s. 6d.) tells the story of our Lord's childhood by the aid of nine coloured illustrations and some conversations on the subject with "Mr. Greybeard." From the Sunday School Union comes "Young England" (5s.), a splendid volume for boys, and "The Child's Own Magazine" (1s.), with an attractive frontispiece, and plenty of nice things for the tiny ones. Wells, Darton and Co. send "Darton's 'Leading Strings'"

(1s. 6d.). This is also a charming book for small children, full of fun and pictures. "Sunday Reading for the Young for 1904" (3s.) is a large volume with suitable and attractive contents for the purpose. Mr. Andrew Melrose's volume "The Girls' Empire" (5s.) is a handsome annual compiled for "English-speaking girls throughout the World.", "Eliza's Husband," by Barry Pain (Chatto and Windus), is too well known to require any introduction. "T. B. B." (Bemrose and Son. 1s.) is amusing and very readable. "Pears' Annual," with three presentation plates and a profusely illustrated narrative of Mons. Dumollet's matrimonial tour, is a wonderful sixpenny-worth.

### Novelties for Nurses.

BY OUR SHOPPING CORRESPONDENT.

### FOR NURSING UNIFORM.

I am pleased to draw the attention of nurses to the specialities supplied by Messrs. Joseph Moore, of Albion Street, Leeds. Our illustrations show two very attractive caps and a neat and becoming bonnet. The caps are made of fine and dainty materials which give them a very superior effect, and the bonnet is equally good in quality and finish. Messrs. Moore supply all varieties of linen goods used in a nurse's uniform. They have caps of different shapes and quality all in good style. These caps are supplied with fine tapes to draw into shape when in use, and lie flat when sent to the laundry. Serviceable and fine aprons, well shaped, are supplied from prices as low as 1s. 6½d. to 3s. 9d. They sell white cap and bonnet strings separately; good white web and linen belts at the low prices of 6½d. and 10½d.; and collars and cuffs in linen and in linenette, a paper material with a linen surface. Messrs. Moore have not long taken up the business of supplying the wants of nurses, but we can confidently recommend them to the attention of our readers, as they evidently know what nurses want and are able to supply it.

### THE "BELLE OF NEW YORK" TOY.

Mrs. Tennyson Harvey, of Wolseley House, 20 St. James's Road, Hastings, wishes it to be known that she will send a pretty and suitable toy for babies to any lady who is collecting articles for a bazaar for charitable purposes. A programme of the bazaar must be enclosed, and mention made that she is applying on account of this announcement in The Hospital. The "Belle of New York" is practically a baby's rattle in the form of a baby doll, dressed entirely in soft Berlin wool, and hung with little bells, surmounted on a short stick which can be grasped by baby hands. It is sure to meet a welcome in the nursery.

### FOR THE SICK-ROOM.

Private nurses have frequently a difficulty in securing suitable and appetising meals for their patients in homes where a bad or indifferent cook is employed. This is especially the case in regard to soups, which are too often greasy and poorly flavoured. Messrs. Cosenza, of 95 Wigmore Street, have practically solved the difficulty by the introduction of their consommé put up into soluble tubes and which we cannot too highly recommend where a light and stimulating soup is desired. The cook cannot spoil the consommé, and the flavour can be varied by the further introduction of vegetables boiled in the water which is used to dilute the consommé, or the excellent vegetable flavouring which Messrs. Cosenza supply in bottles may be added after the consommé is served. The same firm have many other useful and excellent commodities, but none more suitable to invalid diet than the consommé and flavouring.

### DELICIOUS MINCEMEAT.

The making of mincemeat is a wearisome process, where no chopping machine is to be found, and much more of the popular mixture would be used were it not for this. Those who do not care to face the time and trouble entailed should procure some of Messrs. Chivers' mincemeat, sold everywhere. They will find it a most excellent substitute for the home-made article. The blending of flavours is most successful.

### 4711 EAU DE COLOGNE.

Referring to this Eau de Cologne, which was noticed last week, the address of Messrs. Mühlens should be 62 New Bond Street, not 162 New Bond Street.

### Presentations.

Dundee Private Hospital for Women.—Miss Meunieur, on resigning her post as nurse-matron of Dundee Private Hospital for Women, was presented by the committee with a beautiful brass carriage clock, with inscription.

### "The Hospital" Convalescent Fund.

The Hon. Secretary acknowledges with thanks the receipt of £1 from Miss L. J. Attree, 5s. from M. A. Barnes Groom, and 2s. 6d. from Miss E. Jones, Matron of the Home for Invalid Children, Firebeacon Cove, Tiverton.

### Everybody's Opinion.

[Correspondence on all subjects is invited, but we cannot in any way be responsible for the opinions expressed by our correspondents. No communication can be entertained if the name and address of the correspondent are not given as a guarantee of good faith, but not necessarily for publication. All correspondents should write on one side of the paper only.]

### THE NURSE IN SOUTH AFRICA.

"NURSE S." writes: May I be permitted to express my warmest sympathy with the opinions as to the character and behaviour of nurses, individually and generally, set forth in the article headed "The Nurse in South Africa." I would that it were read by and deeply impressed upon the mind of every woman who takes upon herself the solemn, responsible work of tending the sick and dying. With such teaching in its columns THE HOSPITAL cannot fail to prove a great blessing not only to the nursing profession but to the public in general.

### DISCIPLINE AT CHARING CROSS HOSPITAL.

"EIGHT SISTERS" of Charing Cross Hospital write: We sisters of Charing Cross Hospital will feel much obliged if you will be kind enough to grant space for this letter in your issue of this week. Regarding the question of discipline we wish to state that late leave and theatre leave were never granted to any of us by the late lady superintendent oftener than once a week. At the same time we did not understand that we were privileged by right to expect extension of time once a week. We assert that any indulgence granted to us by our late lady superintendent was entirely dependent on her discretion at the moment. Since October, 1903, the present matron has instituted an extension of time—by right—to the sisters to 11.30 P.M. on their day and half-day off; staff nurses, 11.30 P.M. on their day off and 10.30 P.M. on their evening off. With this exception our hours off duty are exactly those quoted by Miss Lucy Rae in her letter of December the 12th.

"MISS E. ALEXANDER" writes: Will you kindly permit me through the columns of your paper to corroborate Miss Lucy Rae's remarks on the discipline of Charing Cross Hospital. For eight and a half years I worked in the institution as probationer, nurse, and sister, and I assert that the nurses were not privileged to ask and obtain late leave once a week as a right. When extension was desired, we all understood that the prerogative of granting or refusing our request rested absolutely on the discretion of the lady superintendent. During my entire term of service discipline was maintained at a very high standard.

### POOR-LAW VACANCIES AND GENERAL HOSPITAL TRAINED NURSES.

"ONE OF THE STAFF" of the Glasgow and West of Scotland Co-operation of Trained Nurses writes: It is a pity, though not a surprise, that "Four Midland Nurses" should display their ignorance to the nursing world by making such a statement as they have done regarding the "testing of urine, giving of hypodermics, and responsibility of important dressings," being so rarely performed by nurses in a general hospital. They do not know what training is in a recognised training school. How can they when their only experience of nursing has been gained in a Poor-law union infirmary? No wonder they feel their position. They would find themselves at sea were they placed in a general hospital and be surprised, no doubt, that points could be given them in the testing of urine, giving of hypodermics, and responsibility of important dressings. I received my training in a well-appointed general hospital, and had experience in all such duties "as most nurses have," it being a part of the training. We have heard a great deal of late about Poor-law infirmaries and the nursing standard being raised. All true nurses will welcome the reform, and I am quite sure that the poor sufferers will hail it as a blessing, so many having been drafted from general hospitals and nursed by women who have been able and permitted to perform in a delicate and refined manner such duties as those quoted by "Four Midland Nurses." I would advise such nurses not to pose as superiors, and remind them that many owe their ability to the teachings and guidance of a hospital-trained nurse.

### THE ANGLO-AMERICAN NURSING HOME AT ROME.

"MISS A." writes: Seeing a statement of the Managing Committe of the Anglo-American Nursing Home in Rome in your paper of December 5th I should like to explain one or two things: To begin with the Committee say "Miss A. still owes us upwards of £30." On this point I beg to differ, as if a statement of my disbursements were made in connection with Nurse X.'s illness it would be seen that I was the creditor rather than the debtor of the Committee. I had to pay at the rate of 12 frs. per day for board and residence at the hotel where we were staying, not mentioning the long delay and the many expenses connected with so severe an illness. Also it was stated that the matron wrote to me for an account that was overdue, but it is necessary to add that the matron sent me an account on April 25th, made up to May 1st. On the following day the nurse fell ill, and I did not consider it necessary to pay for a sick nurse or to settle accounts before they were due. Till the statement of the Committee appeared in your Journal I did not know that the Committee showed any anxiety for a statement of expenses and had they asked for it it would have been placed at their disposal by my solicitor whose advice I have taken. I may mention that I paid to the Anglo-American Nursing Home the sum of over 2,000 frs. (£80), which seems to be a fairly good one for three months' care and for the very inefficient food which was provided. It is true that I had a special nurse instead of being nursed by any nurse who might be "in" and disengaged from private work, and who had then to take her turn and nurse any patient requiring her care—an arrangement unsatisfactory for both patients and nurses alike. The former had the practical experience of a great variety of nurses, and the latter never had the satisfaction of seeing any case to the finish. Also, if anything went wrong none of the nurses were responsible, all more or less incurring blame. I cannot understand why the Committee thought I could dispense with a nurse for I never had the pleasure of a personal visit from any of them, so they were scarcely in a position to judge whether a nurse was required or not. It seems a strange fact that among the list of well-known names which appear at the end of the Committee's statement the name of no doctor is included. One would have expected to have at least seen the name of the doctor who, from the Committee's account, makes so much use of the Home!

"NURSE X." writes: There are a good many things left for me to correct in the statement of the Managing Committee of the Anglo-American Nursing Home in THE HOSPITAL of December 5th.—1. Nurse X.'s case, according to the previous statement, was reported to be a "light one." That may be, but who reported it as such? The doctor, who did not find it necessary to attend to Miss A. for a longer time, in spite of being expected to, or the matron, who came mostly once a day for a few minutes, and then seemed so strangely overworked that at times she even forgot to ask how the patient was? The fact is that the case had been reported many times to both the matron and the doctor, as a heavy one, on account of the patient walking in her sleep nightly for about three months running. That the patient was often fairly well in the day-time and able to go for long drives and amusements, is a fact which, many times goes hand in hand with nerve complaints. 2. On April 24th "Nurse X." asked to be released from her engagement, etc. It is not stated that I mentioned "at the earliest possible convenience, with the first lot of nurses who were supposed to be sent home." The reason I desired to be released was not that I was "wanted at home," but because I had felt ill since the beginning of the month, and longed to go home. 3. A few days later Miss A. telegraphed, etc. Miss A. has never telegraphed once, either to the matron or to anybody, but communicated per telephone with the matron and daily with the doctor in charge. 4. "The doctor, whose identity was not mentioned, had said," etc. That doctor mentioned was an Italian who could not make himself understood in any language except his own, so he said nothing, but prescribed measures similar to those which the matron in Rome prescribed through the telephone on her own responsibility, my temperature then being above 103° Fahrenheit. The doctor who was called in from Rome mentioned distinctly at his second visit to both Miss A. and to me, that he saw the matron and informed her of the nature of the illness as well as of the impossibility of moving me for some considerable time. As for the report that I had "chicken" on May 10th, may the reporter be forgiven, for neither Miss A. nor I set eyes on such a delicacy for the whole of our stay in Tivoli. 5. The certificate given by the medical authority in question, stating that Nurse X had been suffering from gastro-intestinal catarrh, is "not" in the possession of the committee of the Anglo-American Nursing Home, because it is in my hands. A copy of the certificate has been sent to Italy through Miss A., and only one certificate was given throughout the illness. According to the doctor's own words, the expression "gastro-intestinal catarrh" was used in the certificate instead of typhoid fever, so as to avoid any difficulties on behalf of higher charges through the hotel keepers at Tivoli. The medical authority in Germany, who attended me through my severe relapse, has stated the character of my illness with plain distinction as a relapse of typhoid fever, with all the symptoms of that acute disease. 6. "Had Nurse X. returned to the Home," etc. How could I return to the Home and leave my post on my own accord without either being dismissed by my patient or called home by the matron, who knew I felt ill, and when I became severely ill was told so through the telephone, and took no notice of it? Miss A. was forced to take the responsibility, as "18 miles" seemed to be rather a long drive at that time, for the medical adviser of the Anglo-American Nursing Home, or the authority who takes charge of nurses in their employment. 7. Miss A. went to Rome on purpose to inform the matron that she was leaving Tivoli with me the following day. She told two nurses of it, and gave the direct message to one of them, the nurse in charge of the Home. Where in that case is the reliability of the so often mentioned nursing staff?

### POOR-LAW SUPERINTENDENT NURSES.

Mr. Charles Grayson, Master of Ipswich Workhouse, writes: You having on several occasions chosen to honour me with a brief criticism on the various papers and letters I have written on the question of nursing in workhouses, may I ask you to extend to me the favour of allowing me to express through your valuable journal my concurrence with some of the statements contained in what I consider a very practical and sound letter written by "An Irish Superintendent Nurse." She tells us very plainly that in Ireland Poor-law nurses have been for some time independent of the master and matron, and subject only to the medical officer, yet continued friction arises unless the matron and nurse are friendly and considerate to one another. This is exactly what I have again and again repeated, and it is what every experienced and unbiassed person knows to be a fact. Unhesitatingly I say (and I challenge the Workhouse Nursing Association or anyone else to prove to the contrary), that if master, matron, and nurses were left to themselves, there would be no more friction between them than there is between any other officers. I will go further and state that there is really less friction existing at the present time—where the administration is under the master and matron—between the officers in question, than there is between the head official and the nurses of a great many of our separate infirmaries. Surely it cannot be the interference of the uneducated matron in the latter cases that causes the friction. In my opinion the general run of masters and matrons of the present day exhibit far more broad-mindedness and consideration for the comfort of their nurses than the head official does of a separate infirmary, and their restrictions are less arbitrary. I have no desire to be boastful or to unduly court the good graces of nurses when I say that masters and matrons generally are ever ready to recognise the important capabilities of a fully qualified nurse of the present day, and have done everything (though perhaps silently) in their power to enhance the nurse's position notwithstanding all that has been said by some people to the contrary. The only thing that one can blame masters and matrons for, is their apathy in their own defence. It is a poor compliment to pay nurses to try to establish that masters have no regard for them, considering that so many masters, like myself, have chosen their partner in life from the fraternity they are said to have been so arbitrary with. I do not quite agree with "An Irish Superintendent Nurse" that charge nurses are at present generally overpaid, but I again reiterate that there is an undoubted desire on the part of some irresponsible "would-be critics" to establish a lucrative position for those who are in many instances, as the writer states, too profoundly ignorant of domestic economy to undertake the work, and thus by waste and extravagance arouse the displeasure of any conscientious responsible official. There is already a grave tendency to supersede the workhouse infirmary-trained nurse by the appointment of hospital-trained nurses to the best positions in infirmaries. If these ladies are really earnest in their desire to do some good for their fellow-creatures let them first practise domestic duties, afterwards enter the hospital and there have a thorough training, and they then will find plenty of scope for all their energies amongst the sick poor who are struggling for an existence in nearly every town in the kingdom. Such a noble work would win for them and their profession the highest possible praise. Besides, what inducement is there for trained nurses who are dependent on their profession for their livelihood to continue infirmary work if all the best positions are to be secured by those less qualified in many respects than themselves? Although I fully endorse the opinion of "An Irish Superintendent Nurse" with regard to domestic capabilities that should be required of a qualified trained nurse, I cannot agree that workhouse infirmaries are so eminently adapted for older nurses. On the contrary, I think we want bright, intelligent young women who can convey a ray of sunshine and gladness into their wards. There are few aged people, even in their very last days, who do not appreciate and enjoy the cheerful energies of those younger than themselves. Education is an acquisition in any station of life everyone will admit, and in nursing, of course, it is essential. However, it is not altogether book-learning or society manners that a candidate for the nursing profession should possess. She should have a thorough home-training where economy has to be practised to some extent and be the proud holder of the many sterling domestic qualifications mentioned by "An Irish Superintendent Nurse."

### Miss Florence Nightingale and Belfast Nurses.

The following letter was read at the annual meeting of the Belfast Nurses' Home last month:—

10 South Street, Park Lane, W.
November 21st, 1903.

Dear Madam,—Miss Nightingale desires me to say that she has read your letter of the 18th November, and the Report of the Belfast Nurses' Home with great interest, and she congratulates you very much upon the work which is being done. It is her firm opinion that the experience gained by a course of private nursing training is of immense value to the nurses. It develops their self-reliance, resourcefulness, and tact in a way that even the best hospital nursing cannot altogether do; and further, from the patient's point of view, she considers that the moral influence of the nurse, especially in the homes of the very poor, is a power for good that is almost incalculable.

She would like all your nurses to look on that part of their profession as a most precious responsibility, and she sends them her very warmest greetings and good wishes for the success of their great work.

Believe me, yours faithfully,
Alice Cochrane, Secretary.

The Hon. Secretary, Belfast Nurses' Home.

### Echoes from the Outside World.

### Movements of Royalty.

The King, who concluded his visit to Lord Iveagh at Elveden Hall on Saturday, returned to London in the afternoon, attending the Criterion Theatre in the evening. His Majesty spent Monday at Buckingham Palace, and on Tuesday went to Windsor to enjoy some shooting. The Queen, accompanied by Princess Victoria, arrived in London from Sandringham on Tuesday afternoon.

The Prince and Princess of Wales left town on Monday afternoon for Brocket Hall, Hatfield, on a visit to Lord and Lady Mount Stephen. On Saturday the Prince of Wales inspected, at Marlborough House, the first portion of the trophies and prizes given in Great Britain for competition at the Australian Fire Brigade Jubilee Celebration to be held next March. His Royal Highness showed great interest in the work of the Fire Brigade, and said that it had given him pleasure to see the prizes before they were shipped to Australia.

The Duchess of Albany, accompanied by Princess Alice of Albany, visited Kingston-on-Thames last week for the purpose of unveiling two memorials, one being to the late Queen. This memorial takes the form of a terra-cotta statue which has been placed at the head of the main staircase and represents Queen Victoria in her Coronation robes, having the simple words on the base "Victoria, Regina et Imperatrix, 1837-1901."

### Fire at Sandringham.

Early on Thursday morning last week the Hon. Charlotte Knollys awoke about five o'clock with smoke in her bedroom, which is situated above that of Queen Alexandra. She at once roused the household, and her Majesty left her bedroom without delay. The outbreak was due to the concrete bed of the stove in Miss Knollys' room not being sufficiently thick, so that the heat of the fire had ignited the beam beneath, and when discovered was within a few inches of Miss Knollys' bed and was rapidly spreading to the ceiling of the Queen's bedroom. As the result of the celerity displayed by the household fire brigade—who, ever since the disastrous fire at the royal residence in November, 1891, has, by the King's orders, been kept always thoroughly equipped and well practised in their work—the conflagration was speedily subdued, but not before much damage had been done to the two bedrooms. In consequence of the water thrown on the floor of Miss Knollys' room the ceiling of the Queen's bedroom fell in shortly after she vacated it, and some valuable photographs and personal treasures have thus been injured. The damage is estimated at £1,000. The King who was at Elveden on Saturday, at once went to Sandringham to see the extent of the fire. Congratulations to the Queen on her escape unharmed have been sent to her from various parts of the country, including one from Alexandra nurses, to whom her Majesty despatched a telegram of thanks.

### A Famous Philosopher.

The remains of the late Mr. Herbert Spencer were cremated at Golder's Green Crematorium on Monday morning, and the ashes were subsequently privately interred in a tomb at Highgate. In accordance with the special instructions which Mr. Spencer left behind him as to the disposal of his body, there were no flowers, and no one wore mourning at his funeral. With the death of Herbert Spencer there has passed away a famous philosopher and a profound thinker. It was in 1862 that he commenced his monumental work the "System of Synthetic Philosophy," which fills ten large volumes, and he subsequently established his reputation as the foremost scientific scholar of the age. To the unification of knowledge, the co-ordination of all the sciences in a single whole of which they should be the parts, the application of the universal law of evolution to all classes of phenomena whether physical, moral, or social, he devoted a long life of incessant labour and profound thought.

### The Scottish Antarctic Expedition.

It was announced on Tuesday that Mr. Bruce, the leader of the Scottish Antarctic Expedition which was sent out last year on board the *Scotia* had arrived, on December 13th, at Monte Video, Uruguay, from the Falkland Islands. Mr. Bruce reports that all is well in the *Scotia*, and that six men have been left behind in charge of a meteorological station at Cape Pembroke. Mr. Bruce stated before he sailed from Scotland that on leaving the Falkland Islands he would proceed in an easterly direction to the Sandwich group in the Weddell Quadrant and, after investigating these islands, would point southward to as high a southern latitute as was compatible with the best interests of scientific research.

### Women and Tariff Reform.

It is announced that a Women's Section of the Tariff Reform League has been formed, "in the belief that it will materially assist in bringing the national policy advocated by Mr. Chamberlain into every household for discussion and inquiry." Sir Gilbert Parker makes an appeal to the women of the country to join this association, which it will be their own work and office to develop, and to send their names to the organising secretary of the Tariff Reform League, Women's Section, 7 Victoria Street, S.W. The subscription is nominal. The first meeting of the new section was held on Monday at the residence of Lord Glenesk. An executive committee was formed with Mrs. Herbert Chamberlain as president.

### Outrage on a Railway.

A young lady of Stoke-on-Trent, Miss Eva Goss, daughter of a magistrate in the neighbourhood, was the victim of a desperate outrage in a second-class carriage upon the railway last week. She had been to visit relatives at Alsager, near Crewe, and about 7 o'clock in the evening entered the train to return to her home. She was alone when the train left the station, but just as it was nearing the Harecastle tunnel, she was startled to see a man on the footboard on the offside of the train. The door was not locked, and the man opening it entered the compartment, remarking that he had had a narrow escape of missing the train. He appeared to be a working man of about 30 years of age. By his strange behaviour the young lady speedily realised that she was in considerable danger, and endeavoured to reach the communication cord, even succeeding in pulling it slightly; but before she could do more, the man sprang upon her and treated her with the utmost violence. She struggled bravely, but, as her strength failed, she made a final entreaty to the man to take her purse and jewels and leave her alone. This he did, and nothing more has been heard of him, but it is thought by many that the assailant was an escaped lunatic for whom the warders of Cheddleton Asylum have been searching. Miss Goss, though still suffering much from shock, is slowly getting better.

### Exhibition of Postage Stamps.

There is an exhibition of great interest to philatelists to be seen at the Albemarle Galleries, consisting of a collection of postage stamps valued by the owner at £25,000. The gem of the collection is an unused specimen of the dark-blue wood block fourpenny triangular Cape of Good Hope, which is said to be worth £250. An English 9d. stamp of 1862, with hair lines, is valued at £100. A unique block of Mafeking besieged stamps which were sent over by Lady Sarah Wilson, is among other numerous attractions to collectors.

### Notes and Queries.

**FOR REGULATIONS SEE PAGE 135.**

*Over Thirty.*

(105) Can you tell me of a co-operation in London for nurses over 30? I heard that one was about to be organised some time ago.—*I. O. L.*

Do you refer to the Auxiliary Nurses' Society which has been formed comparatively recently for nurses considerably over 30 who belong to the Royal British Nurses' Association? If so apply to the Hon. Lady Superintendent, 10 Orchard Street, W.

*Home.*

(106) Can you tell me of a home or hospital where a blind boy of 10, with dorsal curvature, could be received? His parents are alive, but they could only afford to pay 1s. weekly.—*Matron.*

Write and ask advice from the Secretary of the Invalid Children's Aid Association, 8 Henrietta Street, Covent Garden, Strand, W.C.

Is there an hospital at Margate where a little boy suffering from suppurating glands in the neck could be taken? His mother is very poor and could not afford to pay anything.—*Miss J.*

The Royal Sea Bathing Hospital, Margate; office, 30 Charing Cross, London, S.W. Admission is free if by Governor's recommendation.

*Housekeeping.*

(107) I would be obliged if you could tell me how to obtain a certificate for housekeeping. I am a trained nurse, but it seems impossible to obtain any good post as matron without holding some certificate for housekeeping.—*B. D. B.*

There are numerous schools for domestic economy, Miss Ella Pycroft, 116 St. Martin's Lane, W.C., is the organising secretary for such under the London County Council, and she would doubtless tell you the best and cheapest way to obtain a certificate for housewifery.

*Hospital Training.*

(108) *C. B. M.* and *L. B. B.* wish to know if, after having been trained and certificated at a children's hospital, they are eligible for general training in a London hospital.

Certainly.

*Royal British Nurses' Association.*

(109) Will you tell me where I must apply in order to become a member of the Royal British Nurses' Association?—*A. C. H.*

The Secretary, 10 Orchard Street, W.

*Massage.*

(110) I should be much obliged for the particulars and terms of learning massage.—*M. M.*

The Secretary of the Incorporated Society for Trained Masseuses, 12 Buckingham Street, Strand, W.C., would be able to advise you if you send full particulars of your requirements.

*Elastic Stocking.*

(111) Can the Editor suggest some way of keeping an elastic stocking from slipping down over the knee? I have tried braces and other things, but none is of any use.—*G.*

Have you tried ordinary suspenders? Either buy a pair, or make a broad, comfortable belt, and place two elastic pendants in front so that they give support to the stocking.

*Maternity Work.*

(112) Can you tell me of any nursing institution in London of which a certified maternity nurse without general training could become a member, and receive her earnings by paying a commission?—*A. M. P.*

Possibly you might be able to join some private concern. The more important associations require further qualifications.

### Standard Nursing Manuals.

"The Nursing Profession: How and Where to Train." 2s. net; 2s. 4d. post free.

"Nursing: Its Theory and Practice." (Revised Edition). 3s. 6d. post free.

"Elementary Anatomy and Surgery for Nurses." By William McAdam Eccles, M.D.Lond., M.B. 2s. 6d. post free.

"Elementary Physiology for Nurses." By C. F. Marshall, M.D., B.Sc., F.R.C.S. 2s. post free.

"Ophthalmic Nursing." By Sydney Stephenson, M.B., F.R.C.S., 3s. 6d. net; 3s. 10d. post free.

"Nursing in Diseases of the Throat, Nose, and Ear." By P. Macleod Yearsley, F.R.C.S.Eng., M.R.C.S. 2s. 6d. post free.

"Fevers and Infectious Diseases." 1s. post free.

### For Reading to the Sick.

### CHRISTMAS DAY.

To-day is a day of joy, and the words of an old hymn are ringing in our hearts:—

Hark! the herald angels sing
Glory to the new-born King,
Peace on earth, and mercy mild,
God and sinners reconciled.
Joyful, all ye nations, rise,
Join the triumph of the skies;
With the angelic hosts proclaim,
Christ is born in Bethlehem.

That is the joy, the strength of Chrismas—Christ is born, Christ our God. We need not try to bear the hard times, the sickness, the pain, alone; Jesus is with us, "Jesus our Emmanuel." We know what a help it is in trouble, if a friend comes to stand by us; in pain, if someone we love puts a strong arm round us to hold us up. We are sick and lonely, and away from home; but a Friend is standing by us, who has come from heaven to help us, Jesus, our "God with us." He waits to turn all the pain into pleasure, all the sorrow into joy, if we will only ask Him. Let us take courage, and speak to Him; let us say, "Oh, Jesus, my Saviour, bring Christmas into my heart."

*B. Waugh.*

Thirty years quite veiled from us, except in two sentences: "He went down with them and came to Nazareth, and was subject unto them." "And Jesus increased in wisdom and stature, and in favour with God and man."

Subjection; and growth. The holy life developing like a lovely flower, ripening into perfect fruit, and winning love and honour from all around, the time of conflict not being yet come. Growth, through subjection to the Divine order and the law of life, living in each stage of life, the life of that stage, instead of restlessly struggling into the next stage or striving to prolong the past.

*W. H. Hutchings.*

Jesus, who was foretold by Isaiah as the Prince of Peace; Jesus, over whose Advent the angels sang their song of peace, came to be "our peace" because He came to reconcile us to our Father in Heaven. He came not only to make peace between us and our offended God; by taking away sin by the sacrifice of Himself, He came also to shed abroad His peace in our hearts and lives. He came to rule in the midst of His, and our enemies; and as His dominion advances, so does peace increase.

*R. C. L. Brown.*

Centuries back the shepherds saw
Angel hosts, with joyful awe;
Learned the tidings of the birth,
Heard the message—Peace on Earth:
Babe of Bethlehem, Prince of Peace,
May Thy kingdom still increase.

Age on age has rolled away
Since that earliest Christmas Day;
Still, through all the stretch of time,
Choirs shall sing and bells shall chime:
Babe of Bethlehem, Lord of Love,
Make this earth like Heaven above.

*H. Rose.*

## Figures and Tables

**Figure f1:**
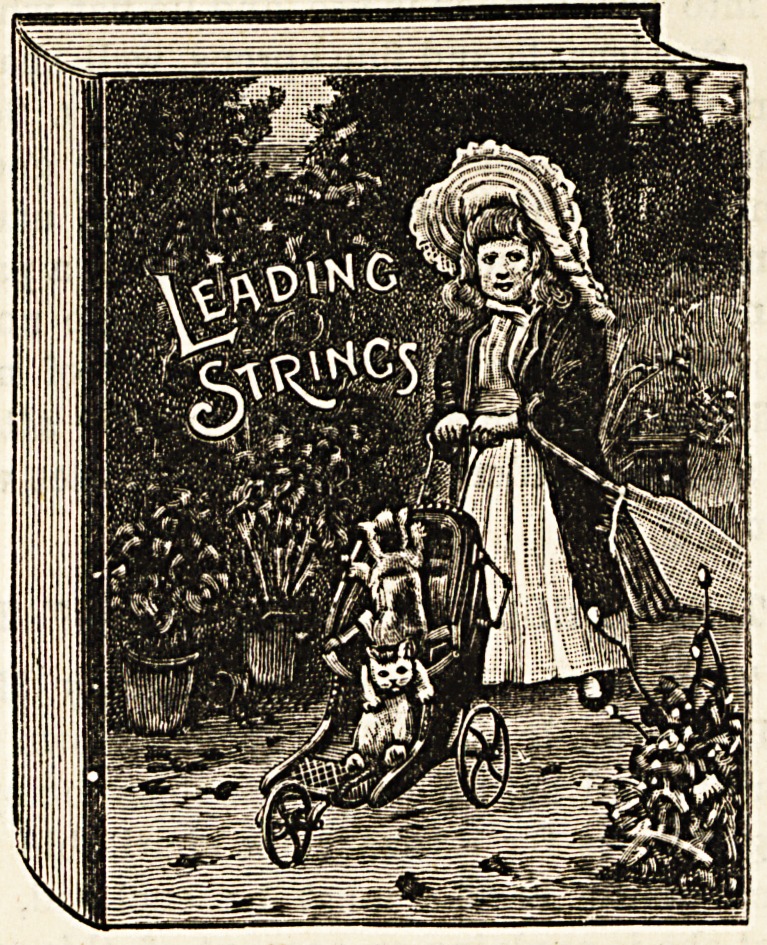


**Figure f2:**
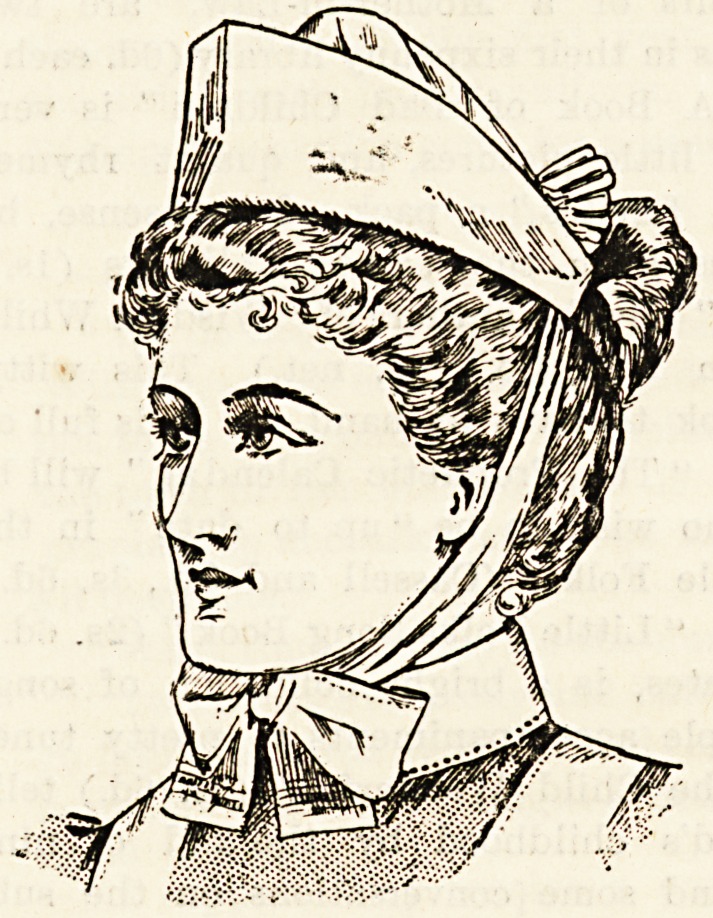


**Figure f3:**
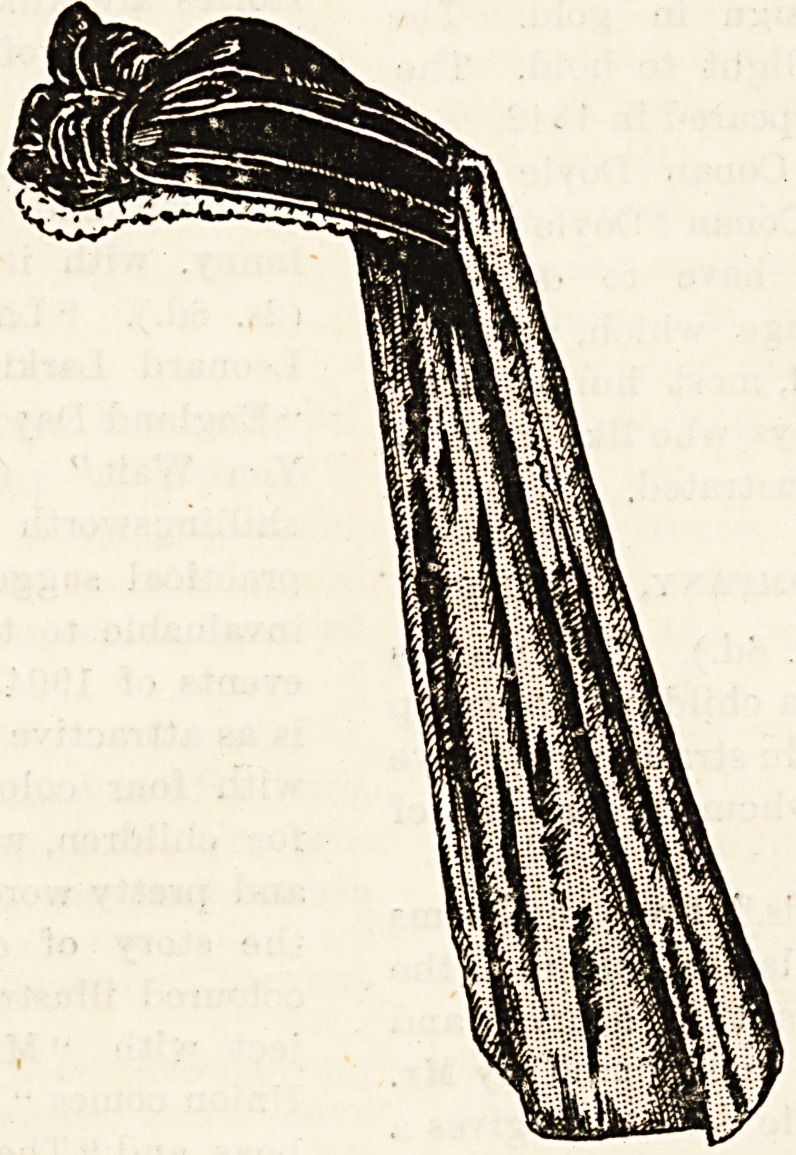


**Figure f4:**